# Evaluation of efficacy and safety of gefitinib as monotherapy in Chinese patients with advanced non-small cell lung cancer and very poor performance status

**DOI:** 10.1186/1756-0500-1-102

**Published:** 2008-10-28

**Authors:** Zhong Wei, Wang Mengzhao, Zhang Li, Li Longyun, Zhang Xiaotong

**Affiliations:** 1 Department of Respiratory Diseases, Peking Union Medical College Hospital, Peking Union Medical College and Chinese Academy of Medical Sciences, Beijing 100730, PR China

## Abstract

**Background:**

This paper reports the outcome of gefitinib for Chinese advanced NSCLC patients with poor performance status (PS) at the Peking Union Medical College Hospital.

**Methods:**

From Oct 2002 to Apr. 2006, 42 advanced NSCLC patients with PS 3/4 received gefitinib 250 mg/day treatment. Median survival (MS) were calculated using the Kaplan-Meier method and a Cox regression model was used to find main factors affecting MS.

**Results:**

Adverse events (AEs) were generally mild (grade 1 and 2) and reversible. The most frequent AEs were rash 72.2% (26/42) and diarrhea 44.4% (26/42). The objective tumor response rate and stable disease rate were 40.5% and 26.2% respectively, and median survival(MS) of all patients was 10.1 months (95% confidential interval CI, 3.4 ~ 16.8), and progression-free survival(PFS) was 5.7 months (95% CI, 4.5 ~ 6.9). The MS were significantly related with objective response of gefitinib. Objective responses was significantly related with rashes induced with gefitinib.

**Conclusion:**

Our study suggest that treatment with gefitinib may be well tolerated and beneficial for Chinese patients with poor PS, and the safety and efficacy were similar to patients with good PS.

## Background

Lung cancer is the leading cause of cancer deaths worldwide. Platinum-based chemotherapy can improve the survival and quality of life for locally advanced and metastatic lung cancer, and the median survival (MS) is about 8 months[1]. Single agent chemotherapy is recommended for patients with an Eastern Cooperation Oncology Group (ECOG) performance status (PS) of 2 and only best supportive care for patients with ECOG PS worse than 2 because of toxicity of chemotherapy. Epidermal growth factor receptor (EGFR) is important in the growth, metastasis, and angiogenesis in NSCLC. Gefitinib (Iressa) is a HER1/EGFR-tyrosine kinase inhibitor for treating patients with non-small cell lung cancer (NSCLC)[2,3]. Two large randomized phase II trials proved the efficacy of gefitinib in pretreated NSCLC patients after relapsing or failing to chemotherapy, with response rates ranged between 10–18.4%[4,5]. As gefitinib has a good safety profile, it had been used in the treatment of patients with ECOG PS of 3–4. Current data show that the efficacy of gefitinib differs much among people of different ethnic origin, and in this paper, we retrospectively reviewed the efficacy and safety of gefitinib in NSCLC patients with PS 3–4 at Peking Union Medical College Hospital in China.

## Methods

### Patients

We surveyed all the patients with NSCLC treated with gefitinib between October 2002 and October 2004 at Peking Union Medical College Hospital. Patients must be 18 years and older with cytology/histopathology-confirmed NSCLC and clinical stages IIIb and IV, and with ECOG PS of 3–4 not fit for surgery, radiotherapy or chemotherapy. Other eligibility criteria included: adequate bone marrow function (absolute neutrophil count > 1.5 × 10^9^/L, platelet count > 100 × 10^9^/L and hemoglobin level > 8.0 g/L), proper liver function (total bilirubin < 1.5 fold of the upper limit of normal value, aspartate aminotransaminase (AST) and alkanine aminotransferase (ALT) < 2.5 fold of the upper limit of normal value), and adequate renal function (serum creatinine < 1.5 mg/dl, blood urea nitrogen < 20 mg/dl). Exclusion criteria included: uncontrolled central nerves system metastases, severe underlying cardio-pulmonary diseases including interstitial pneumonia, habitual diarrhea or constipation and other GI disorders affecting drug absorption. All patients came from clinical trial "Iressa Expanded Access Program (EAP)", which was approved by United States Food and Drug Administration (ClinicalTrials.gov Identifier: NCT00034879). All patients must have written informed consent form.

### Study protocols

One oral gefitinib tablet (250 mg) was taken at about the same time each day without interruption till the occurrence of unacceptable toxicity, disease progression or death. Baseline evaluation was performed within 21 days prior to enrollment, including complete medical history and physical examination, laboratory tests (whole blood counts, urine analysis, liver and renal functions), electrocardiogram, thorax computed-tomography (CT) scan, radionuclide bone scan. The primary end point was survival, and the secondary end points were objective response rate according to the RECIST criteria and progression free survival (PFS). Changes in 3 key symptoms related to lung cancer, including cough, dyspnea and pain, were also recorded. All adverse events were recorded according to National Cancer Institute expanded common toxicity criteria(CTC), version 3.0.

### Statistical analysis

Median overall survival (MS) and median PFS were calculated using the Kaplan-Meier method and a Cox regression model was used to find main factors affecting MS. The factors considered for inclusion in the analyses were age, sex, smoking status, ECOG PS, histology type, bone metastasis, brain metastasis, liver metastasis, pleural effusion, number of previous chemotherapy regimens, response condition and rash inducing by gefitinib. Multiple linear regression model was used to analysis factors affecting tumor response rate (RR). The factors considered for inclusion in the analyses were age, sex, smoking status, histology type, bone metastasis, brain metastasis, liver metastasis, pleural effusion, number of previous chemotherapy regimens and rash inducing by gefitinib.

## Results

### Overall efficacy

**Figure 1:**
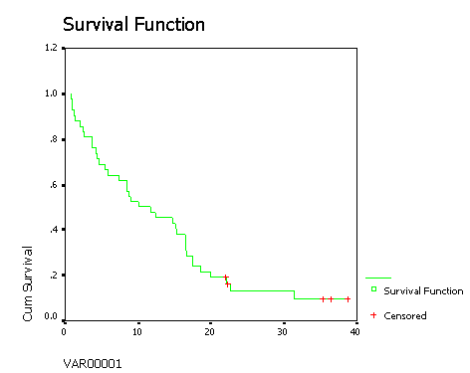
**Survival Function.  **The median overall survival (MS) was 10.1 months (95% CI 3.4–16.8 months), and 1-year survival rate 45%  for advanced NSCLC patients with PS 3/4 received gefitinib treatment.

From Oct 2002 to Apr. 2006, 42 advanced NSCLC patients with PS 3/4 received gefitinib 250 mg/day treatment. The baseline demographic factors are showed [see Additional file 1]. The median overall survival (MS) was 10.1 months (95% CI 3.4–16.8 months), and 1-year survival rate 45% in Figure 1. The median progress free survival (PFS) was 5.7 months (95% CI 4.5–6.9 months). In objective efficacy evaluation, 17 cases (40.5%) had a partial response (PR), 11 (26.2%) stable disease (SD), 7 (16.7%) progressive disease (PD), and 7 (16.7%) no available data.

### Factors that affect survival

Only response to gefitinib had a significant influence on survival through Cox regression analysis [see Additional file 2]. The MS was not related to age, sex, ECOG performance status, histology type, bone metastases, brain metastases, liver metastases, pleural effusion, and prior chemotherapy regimens.

### Factors that affect objective efficacy

It could be seen that only skin rash after treatment were associated with significantly better objective response rate [see Additional file 3]. The objective response rate was not related to age, sex, ECOG performance status, histology type, bone metastases, brain metastases, liver metastases, pleural effusion, and prior chemotherapy regimens.

### Adverse events (AEs)

6 patients were not followed for AEs. Common AEs in 36 patients were listed [see Additional file 4], the most common AE were skin rash, followed by diarrhea and appetite loss. There were very rare grade 3 or 4 AEs. No patients withdrew from gefitinib treatment due to AE.

## Discussion

Chemotherapy has its role in advanced NSCLC patients with good PS, but patients with ECOG PS 3–4 are not suitable for any chemotherapy. Gefitinib is the treatment choice for these special group patients because of the low toxicity. Gefitinib had been used as first line treatment for NSCLC patients with poor PS or refusal to chemotherapy.

The toxicity profile of gefitinib is clearly better than that reported with chemotherapy. The main AEs were skin rash and diarrhea, and most AEs were CTC grade 1 or 2, which was similar to the patients with good PS in IDEAL-1 and IDEAL-2 study [4,5].

In this study, histology type, sex, or smoking status had no difference in objective response, but in some phase II trials, adenocarcinoma, never smokers and female patients had better response rate[6]. This may be due to small sample size or poor PS. In this trial, the objective response rate and disease control rate were 40.5% and 66.7%, respectively, higher than those of 18.4% and 54.4% in IDEAL-1 study, and 11.8% and 42.0% in IDEAL2 study. In IDEAL-1 study, there were 102 Japanese patients, whose objective response rate was 27.5%, similar to our results, suggesting there might be ethnic differences in gefitinib efficacy. Somatic mutations of the EGFR tyrosine kinase were found in 15 of 58(25.9%) from Japan, 28 of 76(36.8%) from China and 1 of 61(1.6%) from the United States[7-9], which may explain the higher response rate in Asia patients. In our patients, the skin rash induced with gefitinib was significantly associated with objective response, and these usually occurs within 2 weeks after treatment, they can be used as indicators for treatment efficacy. Seventy-two advanced NSCLC patients with PS ≥ 2 and no previous chemotherapy were given gefitinib 250 mg per day in USA[10]. Objective response showed PR 4%, SD 46% and PD 26%. PFS and MS were 3.7 months and 6.3 months, respectively. It was drawn that Gefitinib demonstrates modest efficacy and is well tolerated in advanced NSCLC for patients with poor PS. Chang et al[11] reported 52 patients with advanced NSCLC and with PS 3–4 received gefitinib treatment. The response rate was 25.0% and MS was 2.5 months (response group 9.1 months). Adverse events were mainly skin reactions and diarrhea. Single gefitinib is worthy of testing in the future for poor PS patients with advanced NSCLC, especially in Asia.

PS was a significant prognostic factor in predicting survival. In our study, the MS was 10 months for patients with PS 3–4, which was better than best supportive care. The median survival was significantly related with gefitinib objective efficacy only, and no relation with sex, age, smoking status, histology type and prior chemotherapy regimens.

In conclusion, gefitinib has clinically antitumor activity and good tolerability in Chinese patients with advanced NSCLC and poor PS. Formal clinical trial are warranted for these special group patients.

## Competing interests

The authors declare that they have no competing interests.

## Authors' contributions

WZ reviewed all patients regularly and collected their clinical data. MZW participated in the follow-up of patients and performed the statistical analysis.

LZ, LYL and XTZ conceived of the study and participated in its design and coordination. All authors read, contributed to and approved the final manuscript.

## Supplementary Material

Additional file 1 **Patients characteristics.** The data provided the baseline demographic factors of patients. Click here for file

Additional file 2 **Factors affecting survival.** The data provided the results of statistical analysis of factors affecting survival. Click here for file

Additional file 3 **Factors affecting objective response in 35 patients.** The data provided the results of statistical analysis of factors affecting objective response. Click here for file

Additional file 4 **Incidences of main AEs induced by gefitinib.** The data provided the main AEs induced by gefitinib. Click here for file

## References

[B1] Schiller JH, Harrington D, Belani CP, Langer C, Sandler A, Krook J, Zhu J, Johnson DH (2002). Comparison of four chemotherapy regimens for advanced non-small cell lung cancer. N Eng J Med.

[B2] Nakagawa K, Tamura T, Negoro S, Kudoh S, Yamamoto N, Yamamoto N, Takeda K, Swaisland H, Nakatani I, Hirose M, Dong RP, Fukuoka M (2003). Phase I pharmacokinetic trial of the selective oral epidermal growth factor receptor tyrosine kinase inhibitor gefitinib ('Iressa', ZD1839) in Japanese patients with solid malignant tumors. Ann Oncol.

[B3] Cohen MH, Williams GA, Sridhara R, Chen G, Pazdur R (2003). FDA drug approval summary: gefitinib (ZD1839) (Iressa^®^) tablets. Oncologist.

[B4] Fukuoka M, Yano S, Giaccone G, Tamura T, Nakagawa K, Douillard JY, Nishiwaki Y, Vansteenkiste J, Kudoh S, Rischin D, Eek R, Horai T, Noda K, Takata I, Smit E, Averbuch S, Macleod A, Feyereislova A, Dong RP, Baselga J (2003). Multi-institutional randomized phase II trial of gefitinib for previously treated patients with advanced non-small-cell lung cancer. J Clin Oncol.

[B5] Kris MG, Natale RB, Herbst RS, Lynch TJ, Prager D, Belani CP, Schiller JH, Kelly K, Spiridonidis H, Sander A, Albain KS, Cella D, Wolf MK, Averbuch SD, Ochs JJ, Kay AC (2003). Efficacy of gefitinib, an inhibitor of the epidermal growth factor receptor tyrosine kinase, in symptomatic patients with non-small cell lung cancer: a randomized trial. JAMA.

[B6] Kaneda H, Tamura K, Kurata T, Uejima H, Nakagawa K, Fukuoka M (2004). Retrospective analysis of the predictive factors associated with the response and survival benefit of gefitinib in patients with advanced non-small cell lung cancer. Lung Cancer.

[B7] Lynch TJ, Bell DW, Sordella R, Gurubhagavatula S, Okimoto RA, Brannigan BW, Harris PL, Haserlat SM, Supko JG, Haluska FG, Louis DN, Christiani DC, Settleman J, Haber DA (2004). Activating mutation in the epidermal growth factor receptor underlying responsiveness of non-small-cell lung cancer to gefitinib. N Engl J Med.

[B8] Paez JG, Jänne PA, Lee JC, Tracy S, Greulich H, Gabriel S, Herman P, Kaye FJ, Lindeman N, Boggon TJ, Naoki K, Sasaki H, Fujii Y, Eck MJ, Sellers WR, Johnson BE, Meyerson M (2004). EGFR mutations in lung cancer: correlation with clinical response to gefitinib therapy. Science.

[B9] Mu XL, Li LY, Zhang XT, Wang MZ, Feng RE, Cui QC, Zhou HS, Guo BQ (2005). Gefitinib-sensitive mutations of the epidermal growth factor receptor tyrosine kinase domain in Chinese patients with non-small cell lung cancer. Clin Cancer Res.

[B10] Spigel DR, Hainsworth JD, Burkett ER, Burris HA, Yardley DA, Thomas M, Jones SF, Dickson NR, Scullin DC, Bradof JE, Rubinsak JR, Brierre JE, Greco FA (2005). Single-agent gefitinib in patients with untreated advanced non-small-cell lung cancer and poor performance status: a Minnie Pearl Cancer Research Network Phase II Trial. Clin Lung Cancer.

[B11] Chang GC, Chen KC, Yang TY, Yin MC, Lin CP, Kuo BI, Hsu JY (2005). Activity of gefitinib in advanced non-small-cell lung cancer with very poor performance status. Invest New Drugs.

