# Supplementary material for: Evaluation of efficacy and safety of gefitinib as monotherapy in Chinese patients with advanced non-small cell lung cancer and very poor performance status
Source: BMC Res Notes. 2008 Oct 28;1:102. doi: 10.1186/1756-0500-1-102 (PMC2588452; doi:10.1186/1756-0500-1-102)
Supplement: Additional file 1 — Patients characteristics. The data provided the baseline demographic factors of patients. [file 1756-0500-1-102-S1.doc]

Table 1 Patients chararteristics

| Items | No. of patients | % |
| --- | --- | --- |
| Sex |  |  |
| Male | 27 | 64.3 |
| Female | 15 | 35.7 |
| Age |  |  |
| <65 | 35 | 83.3 |
| ≥65 | 7 | 16.7 |
| Smoking status |  |  |
| Yes | 12 | 28.6 |
| No | 30 | 71.4 |
| ECOG PS |  |  |
| 3 | 21 | 50.0 |
| 4 | 21 | 50.0 |
| Histology type |  |  |
| adenocarcinoma | 28 | 66.7 |
| Squamous | 11 | 26.2 |
| Undetermined | 3 | 7.1 |
| Disease stage |  |  |
| IIIB | 1 | 2.4 |
| IV | 41 | 97.6 |
| Bone metastasis |  |  |
| Yes | 19 | 45.2 |
| No | 23 | 54.8 |
| Brain metastasis |  |  |
| Yes | 12 | 28.6 |
| No | 30 | 71.4 |
| Liver metastasis |  |  |
| Yes | 6 | 14.3 |
| No | 36 | 85.7 |
| Pleural effusion |  |  |
| Yes | 13 | 31.0 |
| No | 29 | 69.0 |
| Prior chemotherapy regimens |  |  |
| 0 | 9 | 21.4 |
| 1 | 13 | 31.0 |
| ≥2 | 20 | 47.6 |
