# Supplementary material for: Evaluation of efficacy and safety of gefitinib as monotherapy in Chinese patients with advanced non-small cell lung cancer and very poor performance status
Source: BMC Res Notes. 2008 Oct 28;1:102. doi: 10.1186/1756-0500-1-102 (PMC2588452; doi:10.1186/1756-0500-1-102)
Supplement: Additional file 2 — Factors affecting survival. The data provided the results of statistical analysis of factors affecting survival. [file 1756-0500-1-102-S2.doc]

Table 2：Factors affecting survival

| Items | Exp(B) | 95% CI for Exp(B) | | Wald | P value |
| --- | --- | --- | --- | --- | --- |
| Response to gefitinib |  |  |  | 8.868 | 0.031 |
| SD | 0.587 | 0.232 | 1.483 | 1.269 | 0.260 |
| PD | 1.635 | 0.645 | 4.148 | 1.073 | 0.300 |
| Undetermined | 2.736 | 1.082 | 6.921 | 4.518 | 0.034 |
