# Supplementary material for: Evaluation of efficacy and safety of gefitinib as monotherapy in Chinese patients with advanced non-small cell lung cancer and very poor performance status
Source: BMC Res Notes. 2008 Oct 28;1:102. doi: 10.1186/1756-0500-1-102 (PMC2588452; doi:10.1186/1756-0500-1-102)
Supplement: Additional file 3 — Factors affecting objective response in 35 patients. The data provided the results of statistical analysis of factors affecting objective response. [file 1756-0500-1-102-S3.doc]

Table 3：Factors affecting objective response in 35 patients

| Items | Standardized Coefficients | t value | *P* value |
| --- | --- | --- | --- |
| Age | -0.182 | -1.140 | 0.263 |
| Sex | -0.100 | -0.537 | 0.595 |
| Smoking status | -0.217 | -1.146 | 0.261 |
| Histology type | 0.012 | 0.068 | 0.946 |
| Bone metastasis | 0.050 | 0.290 | 0.774 |
| Brain metastasis | -0.108 | -0.619 | 0.540 |
| Liver metastasis | -0.170 | -1.144 | 0.262 |
| Pleural effusion | -0.143 | -0.898 | 0.376 |
| Prior chemotherapy regimens | 0.034 | 0.220 | 0.827 |
| Skin rash | -0.495 | -2.806 | 0.009 |
