# Supplementary material for: Evaluation of efficacy and safety of gefitinib as monotherapy in Chinese patients with advanced non-small cell lung cancer and very poor performance status
Source: BMC Res Notes. 2008 Oct 28;1:102. doi: 10.1186/1756-0500-1-102 (PMC2588452; doi:10.1186/1756-0500-1-102)
Supplement: Additional file 4 — Incidences of main AEs induced by gefitinib. The data provided the main AEs induced by gefitinib. [file 1756-0500-1-102-S4.doc]

Table 4 Incidences of main AEs induced by gefitinib

| Items | Grade 1（%） | Grade 2（%） | Grade 3（%） | Grade 4（%） | sum（%） |
| --- | --- | --- | --- | --- | --- |
| Skin rashes | 23(63.9） | 3(8.3) | 0 | 0 | 26(72.2） |
| Diarrhea | 13(36.1) | 2(5.6) | 1(2.8) | 0 | 16(44.4) |
| Appetite loss | 3(8.3) | 0 | 0 | 0 | 7(12.5) |
| Nausea/Vomiting | 4(11.1) | 1(2.8) | 0 | 0 | 5(13.9) |
